# The Pace of Cultural Evolution

**DOI:** 10.1371/journal.pone.0045150

**Published:** 2012-09-14

**Authors:** Charles Perreault

**Affiliations:** Santa Fe Institute, Santa Fe, New Mexico, United States of America; Durham University, United Kingdom

## Abstract

Today, humans inhabit most of the world’s terrestrial habitats. This observation has been explained by the fact that we possess a secondary inheritance mechanism, culture, in addition to a genetic system. Because it is assumed that cultural evolution occurs faster than biological evolution, humans can adapt to new ecosystems more rapidly than other animals. This assumption, however, has never been tested empirically. Here, I compare rates of change in human technologies to rates of change in animal morphologies. I find that rates of cultural evolution are inversely correlated with the time interval over which they are measured, which is similar to what is known for biological rates. This correlation explains why the pace of cultural evolution appears faster when measured over recent time periods, where time intervals are often shorter. Controlling for the correlation between rates and time intervals, I show that (1) cultural evolution is faster than biological evolution; (2) this effect holds true even when the generation time of species is controlled for; and (3) culture allows us to evolve over short time scales, which are normally accessible only to short-lived species, while at the same time allowing for us to enjoy the benefits of having a long life history.

## Introduction

Humans dominate the earth’s ecosystems [1]. Today, our uncommonly large range encompasses most of the world’s terrestrial habitats, and human populations thrive in environments as diverse as the Amazonian jungle and the Arctic desert. This adaptive radiation has been explained by our capacity to socially learn information (culture) [2]–[4]. Culture is an inheritance system that parallels and interacts with the genetic system [5]–[8]. Cultural variation and innovations accumulate in a population throughout time, allowing for complex cultural adaptations to evolve [9]–[13]. Because it is assumed that cultural evolution occurs faster than biological evolution on average, humans can adapt to new ecosystems more rapidly than other animals [4]. Yet, the evidence for the hypothesis that cultural evolution is faster than biological evolution is anecdotal [3], [14] and there are no systematic comparisons of cultural and biological rates of change. Moreover, we do not know how much faster, if at all, culture can change compared to biological phenotypes.

Cultural evolution is expected to be faster than biological evolution because of its Lamarckian nature, and because cultural information is transmitted through different routes than genetic information. While variation in biological evolution arises from random mutations, Lamarckian-like guided variation, which occurs through modifications to knowledge, skills and technologies made by an individual that are subsequently transmitted to other individuals, is a potent source of cultural variation [3], [5], [7], [15], [16]. Thus, in contrast to biological evolution, which is blind, cultural evolution can be a directed and consequently faster process. The pace of biological evolution is also constrained by the generation time of the species, since genetic information is transmitted vertically through sexual reproduction. While cultural information can be transmitted from parents to offspring, it is also transmitted obliquely, between non-parents from a previous generation, and horizontally, between contemporaries. This transmission mode gives cultural evolution the potential to spread rapidly in a population, much like an epidemic disease [3], [5], [7], [15], [17].

However, it is not entirely obvious that cultural evolution is faster than biological evolution. On the one hand, the archaeological record is full of instances where traditions have remained remarkably stable over hundreds of years. Microlithic tools, for example, appeared in Northern Asia around 17–18,000 Before Present (BP), and remained part of the hunter-gatherers toolkit until after 14,000 BP [18]. In addition, the Japanese sword, which is a much more complex technology, has been fabricated following essentially the same steps for nearly 700 years [19], [20]. On the other hand, biologists regularly observe evolutionary change over much smaller time scales. Darwin’s Finches, a group of bird species inhabiting the Galapagos Islands, undergo morphological change on a yearly scale in what has become a textbook, classic example of biological evolution [21]. These examples indicate that the distributions of biological and cultural rates of change are, at the very least, overlapping ones. Culture might be less constrained than biology and have the potential to change instantaneously. However, much of what we know from anthropological and psychological research tells us that culture will rarely change instantly. Deviation from a group’s social norms can be costly, and can result in punishment [22]–[25], while social and psychological mechanisms, such as the ones that lead individuals to mark their ethnic identity [26] or conformism [27], will also tend to act against rapid change in an individual’s behavior. Thus, given these forces that can act against cultural change, one can ask what is the characteristic pace of cultural evolution, and how does it compare to the pace of biological evolution? In this study, I try to answer these questions by comparing the rates of change in technologies, as observed in the historical and archaeological record to the rates of morphological change, as seen in contemporary and fossil animal populations.

## Materials and Methods

Biological rates were obtained from previously analyzed data sets [28], [29]. Biological rates are calculated from observations made at various taxonomic levels, ranging from subpopulations of the same species to genus and family. Cultural rates were compiled from the archaeological literature and are described in Table 1. Cultural rates are based on observations made at the level of taxonomic unit that archaeologists often refer to, albeit haphazardly [30] as types, classes, and style; that is, artifacts that share a combination of attributes and that have distinct spatial and temporal distribution. Only rates corresponding to a change in one-dimensional metric attributes were collected. Biological rates observed in a laboratory or human-perturbed settings, as well as those measuring divergence between contemporary sister populations, were excluded. Effort was made to assemble an unbiased sample of cultural rates calculated from what the author(s) of each study interpreted as historically continuous populations. Both biological and cultural rates were measured in *darwins* (*d*), which is a standardized unit of change in factors of *e*, the base of the natural logarithm, per millions of years.
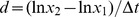
where *x_1_* and *x_2_* are the mean trait value at time 1 and time 2, respectively, and Δ*_t_* is the time interval between *x_1_* and *x_2_*, measured in millions of years. The *darwin* is preferred to the *haldane* [31], which is another metric of the rate of evolutionary change, since standard deviations for metric attributes are often not reported in the archaeological literature and because the generation time for cultural evolution is unclear. The rates analyzed in this study were absolute and non-autonomous [28], [32].

**Table 1:** Source of the cultural rates from the archaeological record (dataset available upon request.).

Technological trait	N of Rates	Absolute rate (*d*)
American Bottom Woodland lithic point maximum length [57]	36	110–21895
American Bottom Woodland lithic point maximum width [57]	77	10–74,901
American Bottom Woodland lithic point stem length [57]	36	697–42,713
American Southwest mano length [58]	6	160–12,348
Anasazi pit structure depth [59]	6	1,304–3,529
Annapolis printer type block height [60]	26	7–3,957
Chesapeake pipe stems diameter [61]	3	252–2,424
Colorado fire features diameter [62]	15	44–3,982
Dagger blade length [63]	6	398–9,956
Dagger blade thickness [63]	3	613–1,842
Dagger blade width [63]	6	201–5,753
Delaware lithic projectile point width [64]	11	217–1,596
European farmhouses length [65]	5	959–1,438
Great point metal projectile point length [66]	3	371–12,730
Knife River Indian Villages glass bead size [67]	19	1,263–14,932
Longhouse length [68]	1	4,684
Michigan and Ontario lithic bifaces base width [69]	15	143–1,079
Missouri ceramic vessel thickness [70]	3	62–490
Missouri ceramic wall thickness [71]	16	58–17,153
Missouri Woodland vessel wall thickness [72]	44	86–12,603
Neutral lithic projectile point length [73]	25	420–45,554
Neutral lithic projectile point width [73]	15	118–37,898
New England clay pipe stems diameter [74]	15	3,364–6,372
New York state Iroquoian ceramic vessel thickness [75]	77	28–4,957
Ontario Iroquois longhouse length [76]	6	309–3,659
Pacific Northwest window glass thickness [77]	25	45,53–29,580
Portuguese ceramic vessel rim diameter [78]	1	1,482
Saskatchewan brass, projectile point length [79]	3	1,820–18,950
Saskatchewan iron and steel projectile point length [79]	28	1,161–614,969
Shoshone River valley projectile points perimeter [80]	36	8.92–1,600
Upland Mogollon pithouse depth [81]	4	943–3,187
Upper Mississippi valley ceramic vessel neck diameter [82]	6	572–3,202

Biological rates are clustered by studies, taxonomic units, subpopulations (if relevant), and traits. Cultural rates are clustered by studies, technologies, and traits. A single variable, named *study-series,* was created and assigned the same unique ID number for the rates belonging to the same study/taxonomic unit/population/trait series or the same study/technology/trait series. To test for the interaction between the type of rates (biological or cultural) or rate and time intervals (or rate ages), a linear mixed model of interaction was used. Time interval was assigned as a fixed effect and *study-series* as a random effect using an unstructured covariance matrix. The linear mixed model controls for the fact that rates are unevenly distributed among the different studies.

Comparing cultural rates of change to biological rates that are calculated from various organisms on an absolute time scale allows us to compare the ability of human culture and biological traits to keep pace with a common driver, such as climate change. Historical contingencies can influence evolution [33]–[35], and both biological and cultural traits can be subjected to a wide range of evolutionary forces, including neutral drift and selection [8]. Identifying the precise evolutionary forces acting on biological and cultural traits, and, for instance, analyzing ‘functional’ traits separately from ‘neutral’ traits, is not a trivial task [36], [37]. In order to circumvent this problem and to obtain the typical pace of biological and cultural evolution, I compare large samples of rates representing a wide range of biological and cultural traits that have undoubtedly been subjected to different regimes of evolutionary forces and historical contingencies.

**Figure 1:**
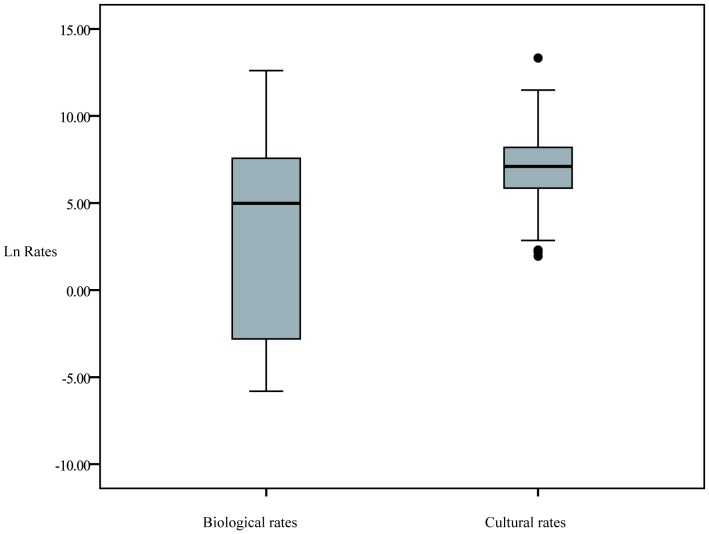
Box-and-whisker plot of the distribution of the absolute values of rates on a natural log scale. On a linear scale, the distribution of biological rates (*n* = 503) has a mean and standard deviation of 3,187±14,457*d*, respectively, and a range of 0.003–298,103.5*d*. The distribution of cultural rates (*n* = 573) has a mean of 4,709±27,069*d* and a range of 7–614,969*d.*

The distributions of biological and cultural rates are significantly different (two-samples Kolmogorov-Smirnov Z = 7.044; two-tailed *P*<0.001). Archaeological rates are on average faster than biological rates, with the fastest cultural rates observed being more than twice as large as the fastest biological rates (Fig. 1).

However, a comparison of rates, as presented in Figure 1, is complicated by the fact that rates of biological evolution are inversely correlated with the time interval over which change is measured [2], [8], [28], [38]–[41]. Evolution appears to operate faster when observing short intervals of time compared to long intervals of time. Two main reasons for this dependency have been suggested [28], [38]–[40], [42]–[45]. First, as the time interval of observation increases, it becomes more likely that the net rate observed is in fact averaged over several disparate rates and evolutionary reversals. This effect is in part driven by the fact that the taxonomic level of the data is likely to increase with the observation time interval. This is true for both biological and cultural evolution. The level of taxonomic units impacts rates of evolution because the evolutionary change that occurs below a taxonomic level (i.e. below the family level) is ignored. Second, rates have the time interval (Δ*_t_*) in the denominator and are therefore proportional to 1/Δ*_t_*. However, because of functional constraints and the effect of stabilizing selection, morphologies rarely change in proportion to Δ*_t_* because they eventually reach evolutionary stasis. In contrast, Δ*_t_* is unbounded and free to vary. Regardless of the mechanisms that drive it, it is necessary to control for the inverse correlation with Δ*_t_* to compare groups of rates. This is accomplished by plotting the rates on an *ln*-rate versus *ln*-time interval graph, and using the linear model that best describes the correlation to compare the groups of rates on the same temporal scale [44].

## Results

**Figure 2:**
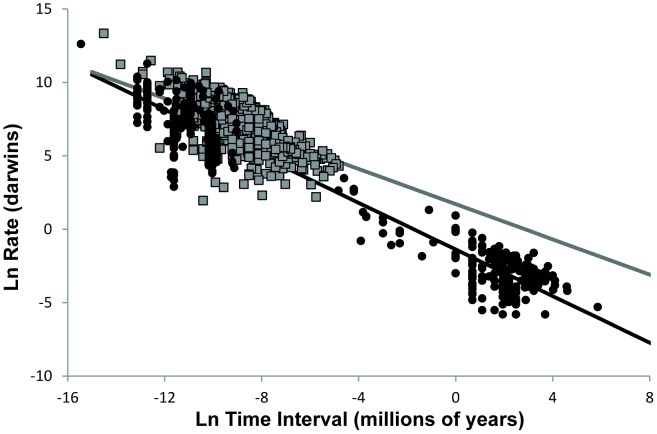
Biological rates and cultural rates plotted against the interval of time over which the rates are measured. Biological rates (black circles) and cultural rates (gray squares) are inversely correlated to the interval of the time over which the rates are measured. The solid lines represent the linear mixed model of interaction that best fit the biological (black line) and cultural (gray line) distributions (Table 2).

**Figure 3:**
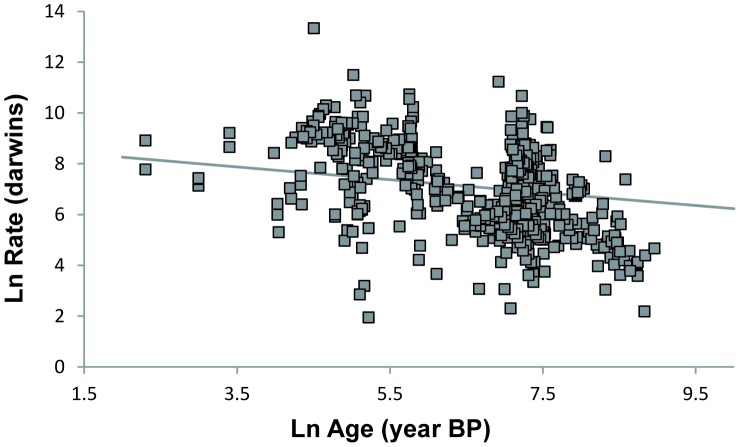
Rates of cultural evolution are inversely correlated with their age. The age of a rate corresponds to the midpoint, in years Before Present (BP), of the time interval over which the rate is calculated. The solid line represents the linear mixed model of interaction of rates with age (Table 3).

A linear mixed model shows that cultural rates are also inversely correlated with the time interval over which they are measured (Fig. 2, Table 2). As we study older periods of our history, taphonomic processes cause the measured time intervals to increase [46]. Cultural rates are therefore inversely correlated with their absolute age (Fig. 3, Table 3). This correlation could potentially account for one of the most salient features of the archaeological record: the pace of change of human material culture appears faster in more recent periods than it does for older ones.

**Table 2:** Estimates of fixed effects and covariance parameters for the linear mixed model of interaction between rate, type of rate (biological or cultural rates), and time interval.

Parameter	Estimate ± S.E.M
Type of rate^1^	−3.088±.455 (*P*<0.001)
Ln (Time Interval)	−0. 599±.01 (*P*<0.001)
Type*Ln (Time Interval)	−0.194±.045 (*P*<0.001)
Intercept	1.71±.45 (*P*<0.001)
Residual	.916±0.051
Variance random intercept	.952±0.121

1 Cultural rates = 0; Biological rates = 1;

The test is performed on the logged (*ln*) values of rates and time intervals. Number of biological rates = 503, number of cultural rates = 573.

**Table 3:** Estimates of fixed effects and covariance parameters for the linear mixed model describing the correlation between rates of cultural change as a function of their age, expressed in years BP.

Parameter	Estimate ± S.E.M
Ln (Years BP)	−.2527±.1 (*P* = 0.021)
Intercept	8.76**±**.1 (*P* = 0.021)
Residual	1.19±0.072
Random Intercept Variance	1.03±0.298

The test is performed on the logged (*ln*) values of rates and ages. Number of biological rates = 503, number of cultural rates = 573.

The difference between cultural and biological rates remains significant even when the effect of the measurement time intervals is controlled (Table 2). We can use the linear mixed model that describes each type of rate to compare them on the same temporal scale. The models converge at the time scale of approximately 1 month (i.e., Δ*_t_ = (1.26×10^−7^)/10^6^ years*), at which point the characteristic rate of change of both biological and cultural change is approximately 76,104 *d*. This corresponds to a ratio between the initial and final value of the attribute undergoing evolution (*x_2_/x_1_*) of 1.01. In other words, at that monthly time scale, both biological and cultural traits will show, on average, a change in their metric attributes of approximately 1%. At the scale of a one-year interval, animal morphologies change at a typical rate of 14,707 *d* (*x_2_/x_1_* = 1.014), compared to 21,989 *d* (*x_2_/x_1_* = 1.022) for archaeological technologies. Cultural change is already recognizably faster at this time scale. At a larger time interval of 1000 years, which is the time scale of millennial climatic fluctuations [41], [47]–[52], the divergence is more pronounced, with morphologies changing at a pace of 60.85 *d*(*x_2_/x_1_* = 1.062) compared to 348.505 *d*(*x_2_*/*x_1_* = 1.4169) for technologies. Thus, cultural evolution is faster than biological evolution when the effects of observational time intervals are controlled. This is due to the fact that for any given time interval, the characteristic amount of accumulated changes (*x_2_ – x_1_*) is greater for culture than for biology. This suggests that cultural change, like biological change, is a multiplicative process: the increments of change in a trait increase as the trait becomes larger.

More surprising, however, is the fact that the magnitude of accumulated cultural changes grows at an increasingly faster pace with time, which is more so than the magnitude of accumulated biological change. Figure 2 shows that the difference between rates of cultural evolution and biological evolution may appear small or even non-existent over a short time scale. However, given enough time, cultural evolution will lead to increasingly larger “morphological” changes than biological evolution, potentially allowing for better accommodation to selective challenges. This result is not an artifact of the bimodal distribution of the time intervals of biological rates. Removing the rates obtained from the paleontological record from the analysis, which account for the rightmost cloud of biological rates plotted in Figure 2, actually increases both the distance between biological and cultural rates as well as the rate at which this difference increases with the time interval (Table 4).

**Table 4:** Estimates of fixed effects and covariance parameters for the linear mixed model of interaction between rate, type of rate (biological or cultural rates), and time interval.

Parameter	Estimate ± S.E.M
Type of rate^1^	−3.98±1.14 (*P* = 0.001)
Ln (Time Interval)	−.6±.04 (*P*<0.001)
Type*Ln (Time Interval)	−.28±.1 (*P* = 0.008)
Intercept	1.7±.46 (*P*<0.001)
Residual	.928±0.054
Variance random intercept	1.29±0.187

1 Cultural rates = 0; Biological rates = 1;

The test is performed on the logged (*ln*) values of rates and time intervals. Rates calculated from the fossil record are excluded from the analysis. Number of biological rates = 283, number of cultural rates = 573.

**Figure 4:**
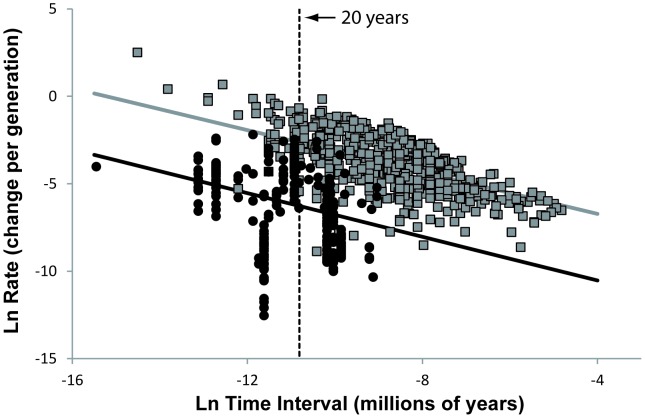
Biological rates and cultural rates calculated as change per generation. Biological rates (black circles) and cultural rates (gray squares), calculated as change per generation, are inversely correlated to the interval of the time over which the rates are measured. The solid lines represent the linear mixed model of interaction that best fit the biological (black line) and cultural (gray line) distributions (Table 5). The conversion of rates from an absolute time scale to a generational time scale impacts mostly the biological rates from species with a short generation time. For any given amount of phenotypic change observed over any given time interval, the difference between rate of change per millions of years and rates of change per generation time increases as generation time of species increases. This effect explains why the difference between the slopes of the two linear models, although significant, is smaller (Table 5) than that shown in Figure 2.

The observation that technologies can change over a short time scale, even if unnoticeably faster than animal morphologies, may be sufficient to make culture adaptive. The minimal time interval at which biological evolution can be observed depends on the generation time of the species. For instance, biological rates calculated over a very short time interval, as plotted in Figure 2, come from species where the life span is counted in months. Several cultural rates have been observed over similarly short time scales, while the generation time of our species is approximately 20 years. Another way to look at this is to calculate evolutionary rates as change per generation, rather than as change per million years. To do this, I have assumed a generation length for humans of 20 years for the computation of cultural rates. The biological rates calculated from the fossil record were also excluded from the analysis, because we do not have good generation time estimates for them. This analysis shows that the pace of cultural change, per generation, is faster than the rate of biological evolution over all observation time intervals (Fig. 4, Table 5), including time intervals that are equal or shorter than the generation time of humans.

**Table 5:** Controlling for the effect of generation time.

Parameter	Estimate ± S.E.M
Type of rate^1^	−3.91±.02 (*P* = 0.01)
Ln (Time Interval)	−.599±.05 (*P*<0.001)
Type*Ln (Time Interval)	−.026±0.15 (*P*<0.001)
Intercept	−9.12±.55 (*P*<0.001)
Residual	.927±0.05
Variance random intercept	3.88±0.43

1 Cultural rates = 0; Biological rates = 1;

Estimates of fixed effects and covariance parameters for the linear mixed model of interaction between rate, type of rate (biological or cultural rates), and time interval. The analysis controls for generation time by calculating rates as the amount of change per generation time (cultural rates are calculated assuming a generation time of 20 years). The test is performed on the logged (*ln*) values of rates and time intervals. Number of biological rates = 283, number of cultural rates = 573.

## Discussion

In this study, I aimed to test the hypothesis that the pace of cultural evolution is faster than the pace of biological evolution. I found that (1) Similar to biological rates, rates of cultural change are inversely correlated with the time interval over which they are measured; (2) this inverse correlation explains, at least in part, why the pace of change appears to be faster for more recent periods of the archaeological record; (3) when controlling for this inverse correlation, rates of cultural evolution are significantly faster than rates of biological evolution; (4) The magnitude of accumulated cultural change grows at an increasingly faster rate with time, which is greater than the magnitude of accumulated biological change. This could mean that some of the adaptive benefits of cultural transmission are not reaped during the short term, but rather occur over longer time scales. However, the fact that biological rates decrease more rapidly with time interval could be driven in part by individual phenotypic plasticity. Phenotypic plasticity is comparable to cultural evolution and allows organisms to change over short time intervals. It is possible that some of the biological rates calculated over a short time interval reflect rapid phenotypic change, while the changes observed over longer time intervals are mostly due to genetic change; and (5) the amount of cultural change observed per generation time (20 years) is significantly faster than what we would expect from biological evolution for a species with the same generation time as humans. This observation highlights the fact that culture allows us to evolve over time scales that are normally accessible only to short-lived species, while at the same time allowing us to enjoy the benefits of having a long life history, such as a large brain, an extended juvenile period, and long life span.

However, it is likely that the archaeological record underestimates rates of cultural change over small time intervals compared to the fossil record. Correct measurements of evolutionary rates must be calculated using phylogenetically-linked populations. Large and rapid changes can obscure phylogenetic relationships and lead to a loss of visibility of the most rapid changes from the data [28]. Fossil species are more likely to retain several homologous traits over longer periods of time compared to culturally transmitted technologies. This may allow for fossils to be compared despite a rapid evolution of other traits. The retention of trait homologies also allows fossils to be compared over greater spatial distances, which increases the chance that they have experienced divergent selective pressure and subsequently a greater morphological differentiation. In contrast, it is often impossible to distinguish rapid cultural evolution in the archaeological record from population replacement. Because of this uncertainty, the cultural rates that were analyzed in this study originated from populations that remained in the same locality, with spatial propinquity being used as a measurement of historical continuity. Instances of rapid cultural evolution over short time scales may therefore be underrepresented in these samples.

Finally, an underlying assumption in this study is that the pace of the change of technologies is as representative of cultural evolution, in general, as the pace of morphological change is representative of biological evolution, which makes the two comparable. Yet, it is unclear as to what extent these findings can be extended to other domains of human cultures, such as social norms, institutions, or political structures, or compared to known rates of change in language [53]–[55] and performance of modern information technologies [56]. While many questions about the pace of cultural evolution remain to be resolved, the study presented here illustrates the utility of an archaeological perspective on patterns of cultural evolution.
